# Gene overlapping and size constraints in the viral world

**DOI:** 10.1186/s13062-016-0128-3

**Published:** 2016-05-21

**Authors:** Nadav Brandes, Michal Linial

**Affiliations:** Einstein Institute of Mathematics, The Edmond J. Safra Campus, The Hebrew University of Jerusalem, Jerusalem, Israel; Department of Biological Chemistry, Room A-530, Institute of Life Sciences, The Edmond J. Safra Campus, The Hebrew University of Jerusalem, 91904 Jerusalem, Israel

**Keywords:** Viral evolution, Open reading frame, Capsid, Icosahedral virion, ViralZone, VIPERdb, Baltimore groups

## Abstract

**Background:**

Viruses are the simplest replicating units, characterized by a limited number of coding genes and an exceptionally high rate of overlapping genes. We sought a unified evolutionary explanation that accounts for their genome sizes, gene overlapping and capsid properties.

**Results:**

We performed an unbiased statistical analysis of ~100 families within ~400 genera that comprise the currently known viral world. We found that the volume utilization of capsids is often low, and greatly varies among viral families. Furthermore, although viruses span three orders of magnitude in genome length, they almost never have over 1500 overlapping nucleotides, or over four significantly overlapping genes per virus.

**Conclusions:**

Our findings undermine the generality of the compression theory, which emphasizes optimal packing and length dependency to explain overlapping genes and capsid size in viral genomes. Instead, we propose that gene novelty and evolution exploration offer better explanations to size constraints and gene overlapping in all viruses.

**Reviewers:**

This article was reviewed by Arne Elofsson and David Kreil.

**Electronic supplementary material:**

The online version of this article (doi:10.1186/s13062-016-0128-3) contains supplementary material, which is available to authorized users.

## Background

Viruses are the simplest biological replicating units and the most abundant ‘biological entities’ known. A great diversity is evident in their physical properties, genome size, gene contents, replication mode and infectivity. Some of the most significant properties of viruses are their small physical size and an exceptional amount of overlapping genes (OGs) relative to their genome length [1, 2]. Most viruses have a high evolutionary rate compared to other organisms [3, 4], with that of RNA viruses 2–3 orders of magnitude higher than DNA viruses [5]. The high mutation rate of RNA viruses is mostly due to the absence of a proof reading mechanism in their replicating enzymes (i.e., RNA polymerase) [6]. It has also been shown that mutation rate is inversely correlated with genome length, not only in viruses [4, 7]. The fast evolution of viruses is dominated by many factors, including their high mutation rate [8], large population size and fast recombination rate [9]. Additionally, their capacity for ‘mix and match’ during co-infection [10, 11] and for hijacking sequences from the host [12] accelerate their evolutionary rate. The non-conventional evolution of many viruses leads to inconclusive and often conflicting theories about their origin [11, 13–15]. Due to the inability to track the full evolutionary history of viruses, their taxonomical hierarchy is fragmented and remains debatable [16].

Viruses are partitioned into seven groups according to their genetic material and replication modes [17]. The two largest groups are double-stranded DNA (dsDNA) and single-stranded RNA (ssRNA+) viruses. In some families the genetic material (RNA or DNA) is segmented and composed of multiple molecules of different lengths. Different genomic segments are often packed into separate virions in the population, and a successful infection is achieved by co-infection [18]. These are collectively called segmented viruses (e.g., Brome mosaic virus, BMV) [19].

All viruses depend heavily on their host’s translation machinery. Only a small set of proteins that fulfill the essential functions for infection are common to all viruses [14, 20]. These functions are restricted to: (i) recognition of the host cell, (ii) replication according to the viral group, and (iii) capsid building.

In a mature virion, the viral genome is encapsulated and protected by a capsid shell, a complex structure built of multiple (usually identical) protein subunits. The most common capsid shape is icosahedral [21], but other structures including rod-like and irregular shapes are also known [22]. An icosahedral capsid is composed of identical elementary protein subunits joined together in a repetitive symmetric pattern. The geometry of icosahedral solids dictates that the number of subunits can take only a fixed set of discrete values (e.g., 60, 180, etc.), determined by a property called the icosahedric triangulation (T) number [23]. In some viruses (e.g., Simplexvirus of the family Herpesviridae), a lipid layer decorated with envelope proteins surrounds the capsid shell [24].

A strong characteristic observed in most viruses is an abundance of overlapping open reading frames (ORFs). Many of these ORFs lack a known function [25]. Overlapping is a universal phenomenon, ubiquitous throughout the entire tree of life, including mammals [26], yet only in viruses it is present in a major scale [27]. Gene overlapping originates from various mechanisms, most notably the use of alternative start codons, ribosomal read-throughs and frame shifts [28]. The tendency for overlapping events is even higher in RNA viruses and in viruses with shorter genomes [29, 30].

Several studies have suggested various explanations for the abundance of overlapping genes (OGs) in viruses. One theory states that since viruses (especially RNA viruses) have a high mutation rate, overlapping events can increase their fitness in various ways [28]. For example, OGs can act as a safety mechanism by amplifying the deleterious effect of mutations occurring within them, thus quickly eliminating such mutations from the viral population [31].

Another theory argues that overlapping has a role in gene regulation by providing an inherent mechanism for coordinated expression. In support of this theory is the presence of OGs that are functionally related or coupled by a regulatory circuit (e.g., a feedback loop) [28, 32].

A third theory describes overlapping as an effective mechanism for generating novel genes, by introducing a new reading frame on top of an existing one [2]. According to this theory, pairs of OGs are usually composed of an old well-founded gene, and a novel gene that was overprinted on top of it [2, 33].

The most accepted theory argues for genome compression as the driving evolutionary force [1, 28, 34, 35]. Multiple arguments were raised to explain the need of viruses to have compact genomes: (i) The high mutation rate of viruses prevents them from having a long genome, as the likelihood of a deleterious mutation in each generation is length dependent [28]. (ii) The advantage for infectivity of shorter genome that lead to faster replication. (iii) The physical size constraint imposed by the capsid’s volume [1]. The physical size constraint is argued to be most dominant in icosahedral viruses due to the discrete nature of the T number, allowing only non-continuous changes in capsid size [34, 36]. Small viruses are also argued to be subject to an even greater evolutionary pressure towards compactness, hence their high abundance of overlapping [37].

Viral evolution is considered at different time scales. The short-range evolution is exemplified by seasonal isolates of influenza strains [38, 39] or HIV-1 variants collected along the progression of the disease [40]. Results from short-term evolution are beneficial for rational treatments [41] and vaccination [42]. In contrast, long-range evolution of viruses is harder to trace. The similarity among viral families in most cases is minimal and below statistical significance.

The motivation for this study is to systematically assess the different theories that aim to explain long-term evolution. We approach this task by an unbiased statistical analysis of the entire viral world. Currently, over 2.4 million viral proteins are archived in the UniProt public database [43]. These proteins belong to viruses from the seven viral groups (and additional 1 % of uncharacterized proteins from metagenomic projects). We took advantage of the high-resolution structural data of some viral capsids [44], and a curated resource for viral classification [45]. This high quality curated database provides a non-redundant representation of reference genomes and proteomes of all known viruses.

## Results

### The landscape of overlapping genes and genome length

Although the subject of gene overlapping has already been extensively studied (e.g., [34]), we present a revised assessment, based on the following considerations: (i) inclusion of all known viruses; (ii) unbiased sampling of the viral space based on well-curated taxa (composed of ~400 genera within ~100 families) as reliable representatives of the viral world; (iii) dealing only with non-trivial overlapping events (i.e., considering segments of protein-coding regions of different ORFs).

**Fig. 1:**
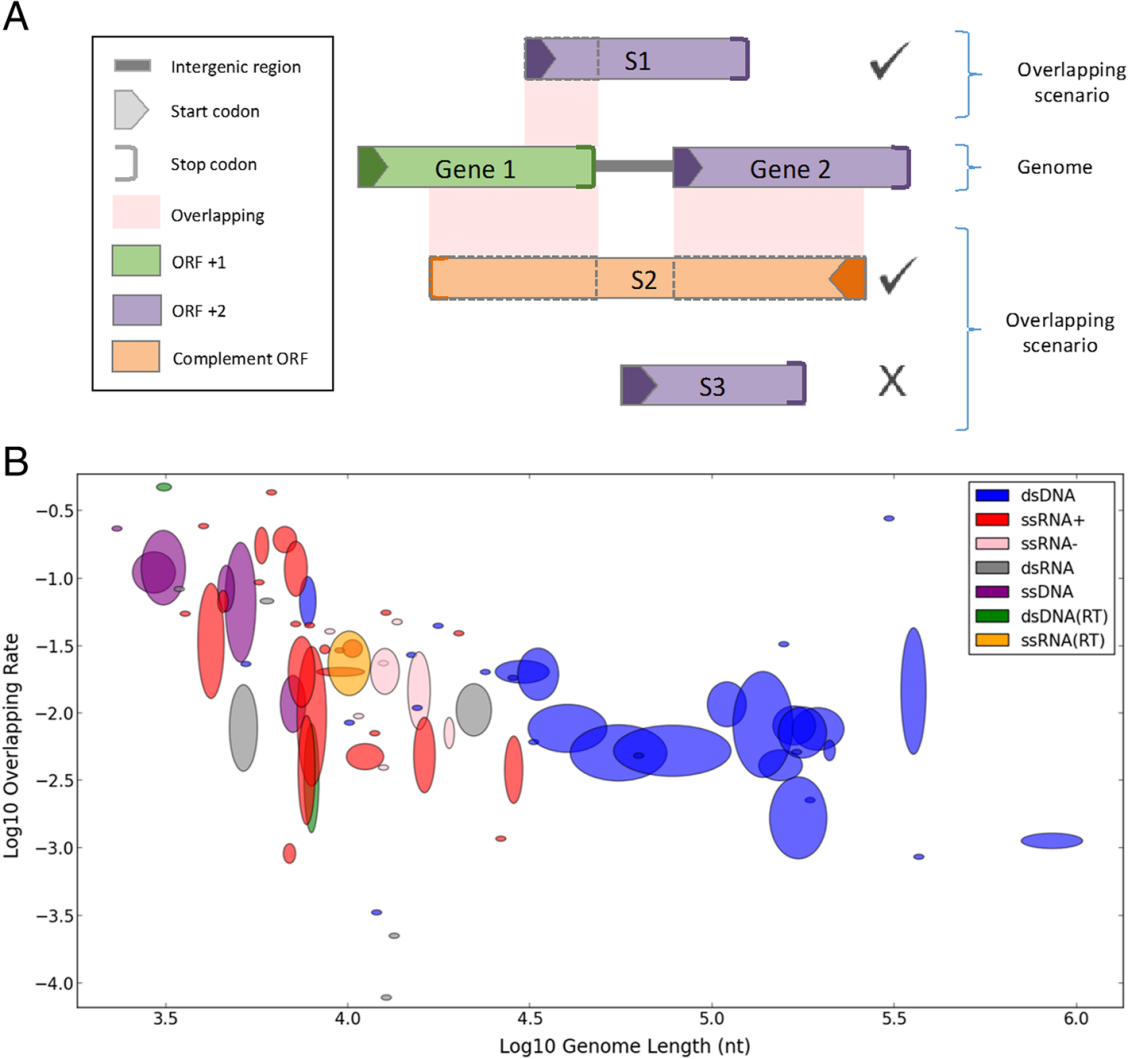
Overlapping rate is negatively correlated to genome length. **a** Illustration of overlapping scenarios. The definition of overlapping in this study is restricted to the presence of two genes that overlap in their coding regions while the other parts of the gene are ignored (e.g., 5′ and 3′ UTRs, or intergenic regions). The same applies for the rare cases of viral genes with introns. We consider only pairs of genes that use different ORFs as overlapping genes. It follows that the first example gene (marked S1) overlaps only with Gene 1, while its “overlap” with Gene 2 that shares the same ORF (frame +2) is not considered (the later is considered a trivial overlap). The second example gene (marked S2) demonstrated that a single gene could participate in multiple overlapping events. The third example gene (marked S3) is not involved in any (non-trivial) overlapping event. The light pink marks the only segments of overlapping. For clarity, we identified each ORF by its own color. **b** A scatter plot demonstrating the negative correlation between genome lengths and overlapping rate in viral families. Both axes are in log scale. 13 families without any overlapping were filtered out (to allow the use of log scale, as had been done in the original work by Belshaw et al. [1] we replicated here ), leaving 80 families out of the complete data set of 93. The families are represented as ellipses, whose width and height correspond to the standard deviation of the genera within them (see Methods). The ellipses are colored by the partition of the families to viral replication groups (see Background). Spearman’s rank correlation: ρ = −0.59, *p*-value = 6.97·10E-9

Figure 1a shows trivial and non-trivial overlapping scenarios. A trivial overlapping event is when the two genes overlap while using the same reading frame (and strand). The rest of the analysis will consider only non-trivial overlapping events (for definition, see Methods). Figure 1b shows that genome length and overlapping rate (i.e., the fraction of the genome involved in overlapping; see Methods) are in a strong negative correlation, as reported before (e.g., [1]), meaning that smaller genomes tend to have higher overlapping rates. This strong correlation (ρ = −0.59, *p*-value = 6.97·10E-9) remains strong when natural partitions of the viral space (e.g., single- or double-stranded viruses) are considered. In all figures, families are represented as ellipses, whose sizes correspond to the variance of the genera within them (see Methods).

**Fig. 2:**
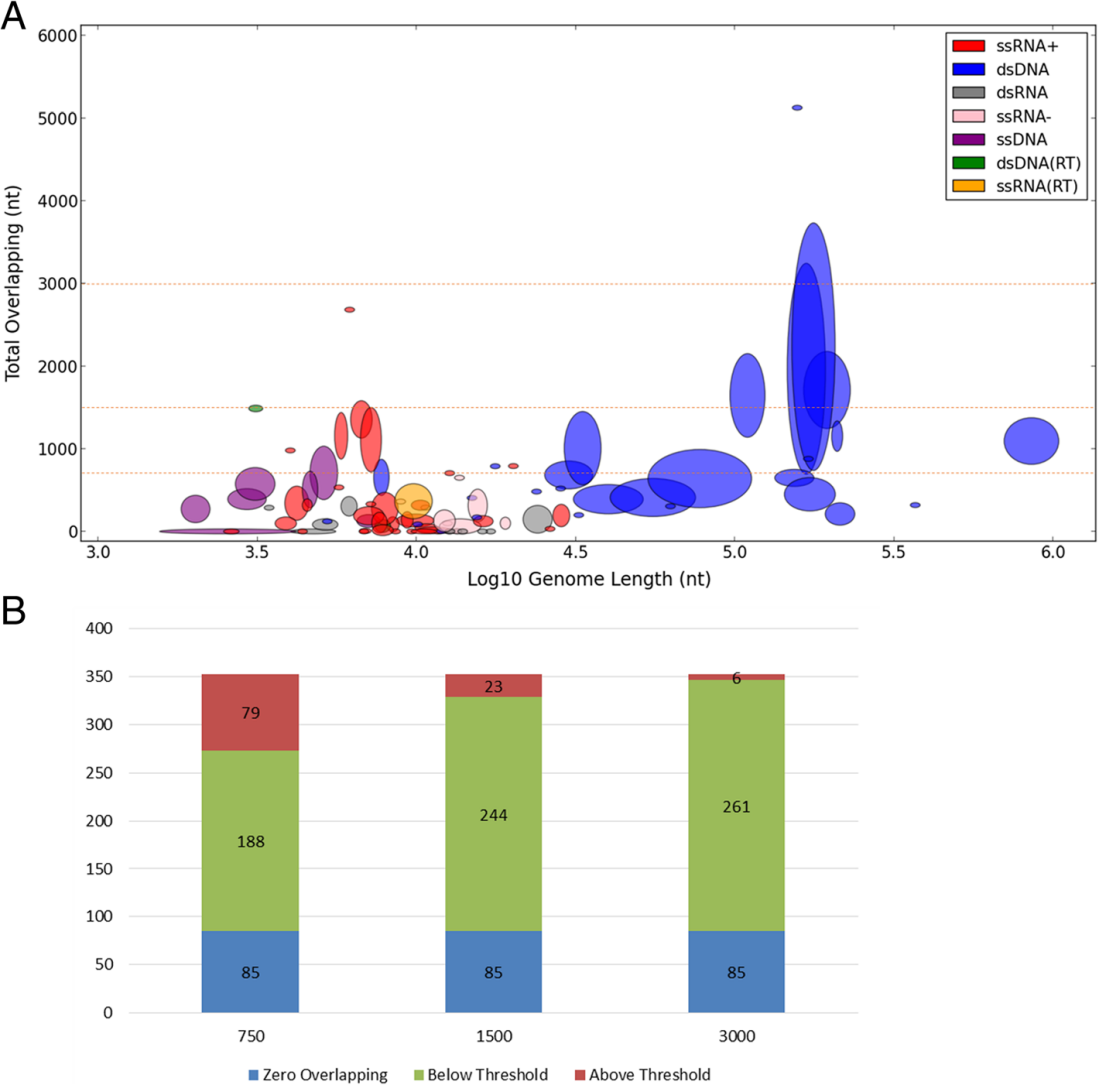
Overlapping amount is strictly bounded. **a** A scatter plot showing the absolute number of overlapping nucleotides and genome lengths of all viral families. Only the X-axis is in log scale. Throughout the entire spectrum of genome length, viral genomes have a bounded amount of nucleotides involved in overlapping. Filtered out 3 outlying families (Nimaviridae, Phycodnaviridae and Iridoviridae with 85,155/305,110, 30,798/357,847 and 7956/144,698 overlapping/total nucleotides respectively), leaving 90 shown families. Spearman’s rank correlation is minimal (ρ = 0.26, *p*-value = 0.015). The dashed lines serve as thresholds (750, 1500 and 3000 nt) that demonstrate the bounded nature of the overlapping amount. Note that most viral families are below these bars. **b** Of the complete data set of 352 genera, most (273, 329 and 346) have a total number of overlapping nucleotides below the chosen thresholds (750, 1500 and 3000 nt), of which 85 genera (24 %) have no overlapping at all. Although the selection of thresholds is somewhat arbitrary, it can be seen that a saturation point is reached at around 1500 nt

A more direct way to measure overlapping is by absolute (rather than relative) amount. Surprisingly, the absolute amount of overlapping (measured in nucleotides, nt) remains highly bounded throughout the entire viral world (Fig. 2), regardless to genome length, which spans across three orders of magnitudes (~1500 to ~1,000,000 nt). The absolute amount of overlapping is bounded by 1500 nt, with only 23 of 352 genera (6.5 %) and nine of 93 families (9.7 %) above this bar. When elevating the bar to 3000 nt, only 6 of the 352 genera (1.7 %) and four of the 93 families (4.3 %) crossed it. Notably, throughout the entire spectrum of genome length, there can be found some families with a close-to-zero amount of overlapping, and other families close to the upper threshold. This is surprising, as one could have anticipated that only the viruses with high genome length will reach the upper bound.

This overlooked observation provides a stronger result than the negative correlation shown in Fig. 1b, which turns out to be merely a byproduct of the relative (rather than absolute) manner in which overlapping rate had been measured prior to our analysis. Specifically, when a more-or-less constant variable (absolute overlapping amount) is divided by a second variable (genome length), the division result will obviously be negatively correlated with that second variable. This is not a byproduct of using different data sets, but a direct outcome of our analysis.

We further tested whether our observation of a natural boundary would remain solid when counting the number of genes (rather than nucleotides) involved in overlapping, as minor overlapping events carry little constraints from evolutionary perspective (see Discussion). We considered only the subset of significantly overlapping genes (SOGs), defined by at least 300 overlapping nucleotides.

**Fig. 3:**
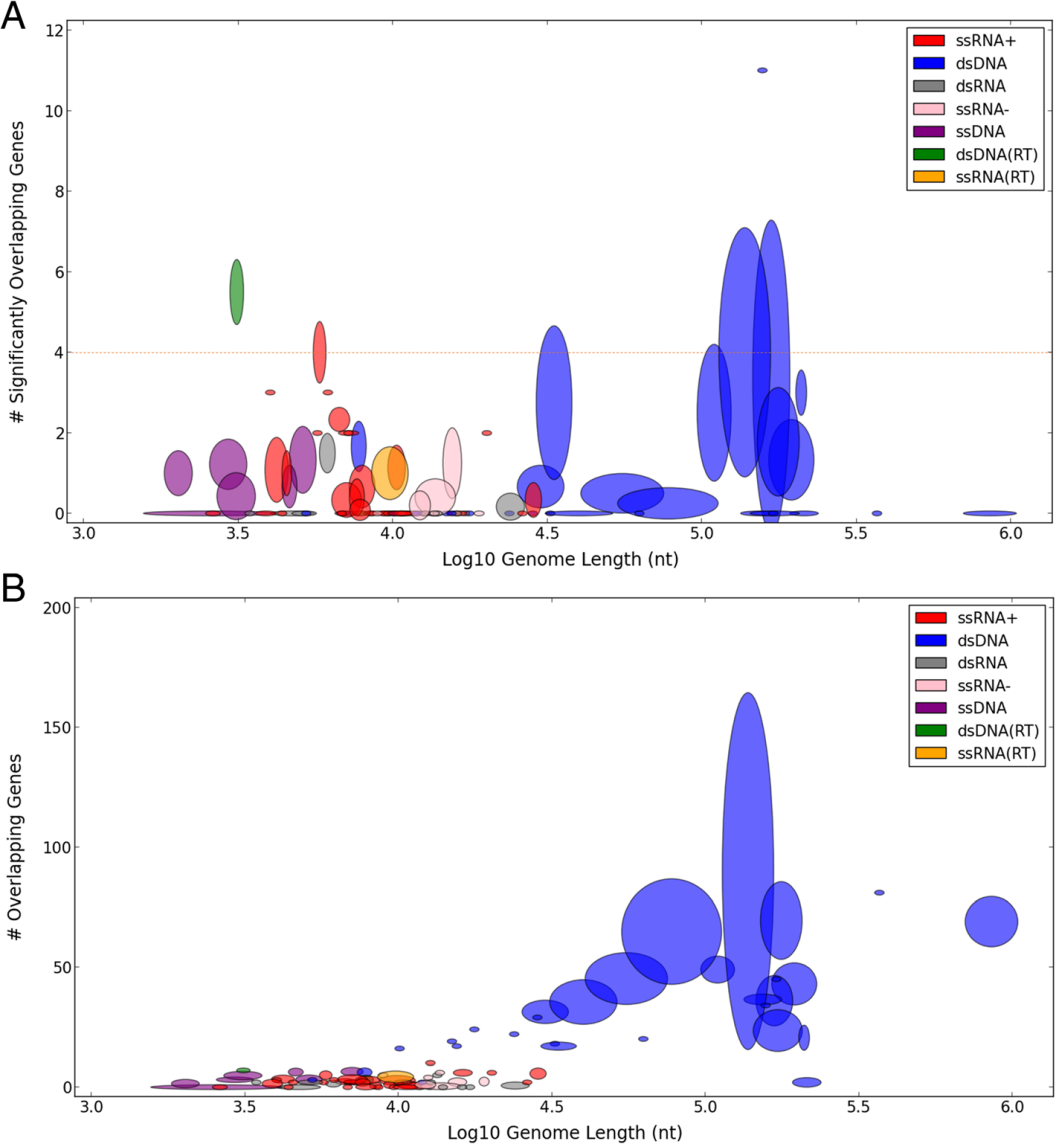
The number of significantly overlapping genes is bounded. **a** A scatter plot demonstrating the number of significantly overlapping genes (SOGs) with respect to genome lengths is shown for 91 of the 93 viral families. Filtered out 2 outlying families (Nimaviridae and Phycodnaviridae with 141 of 532 and 50 of 505 significantly overlapping genes respectively). Only the X-axis is in log scale. Spearman’s rank correlation shows no significance (ρ = −0.08, *p*-value = 0.43). Most families have less than 4 significantly overlapping genes (dashed line), which account for less than 2 gene pairs. **b** A scatter plot demonstrating the number of all overlapping genes when no thresholds is used, with respect to genome lengths. Only the X-axis is in log scale. Filtered out 2 outlying families (Nimaviridae and Phycodnaviridae with 489 of 532 and 283 of 505 overlapping genes respectively), leaving 91 shown families. Spearman's rank correlation: ρ = 0.55, *p*-value = 1.25°10E-8

Figure 3a shows that the number of SOGs also remains highly bounded, with almost all virus families below four such genes, translating to less than two significant overlapping events. Only 3.4 % of the genera and 4.3 % of the families exceed this bound. Importantly, there can be found both very small and very big viruses meeting both the higher (four genes) and lower (zero genes) bounds.

Repeating the same analysis with varying thresholds for SOGs (50 or 100 nt, instead of 300) yields similar results (Additional file 1). However, when the threshold is eliminated altogether and all overlapping events are considered, including very minor ones (of only a few nucleotides) the total number of OGs steadily grows with genome length (Fig. 3b). Since the number of SOGs remains stable, it can be deduced that only minor overlapping events become more abundant in bigger genomes (Spearman’s rank correlation: ρ = 0.55, *p*-value = 1.25·10E-8).

### Overlapping is not associated with virion shape

**Fig. 4:**
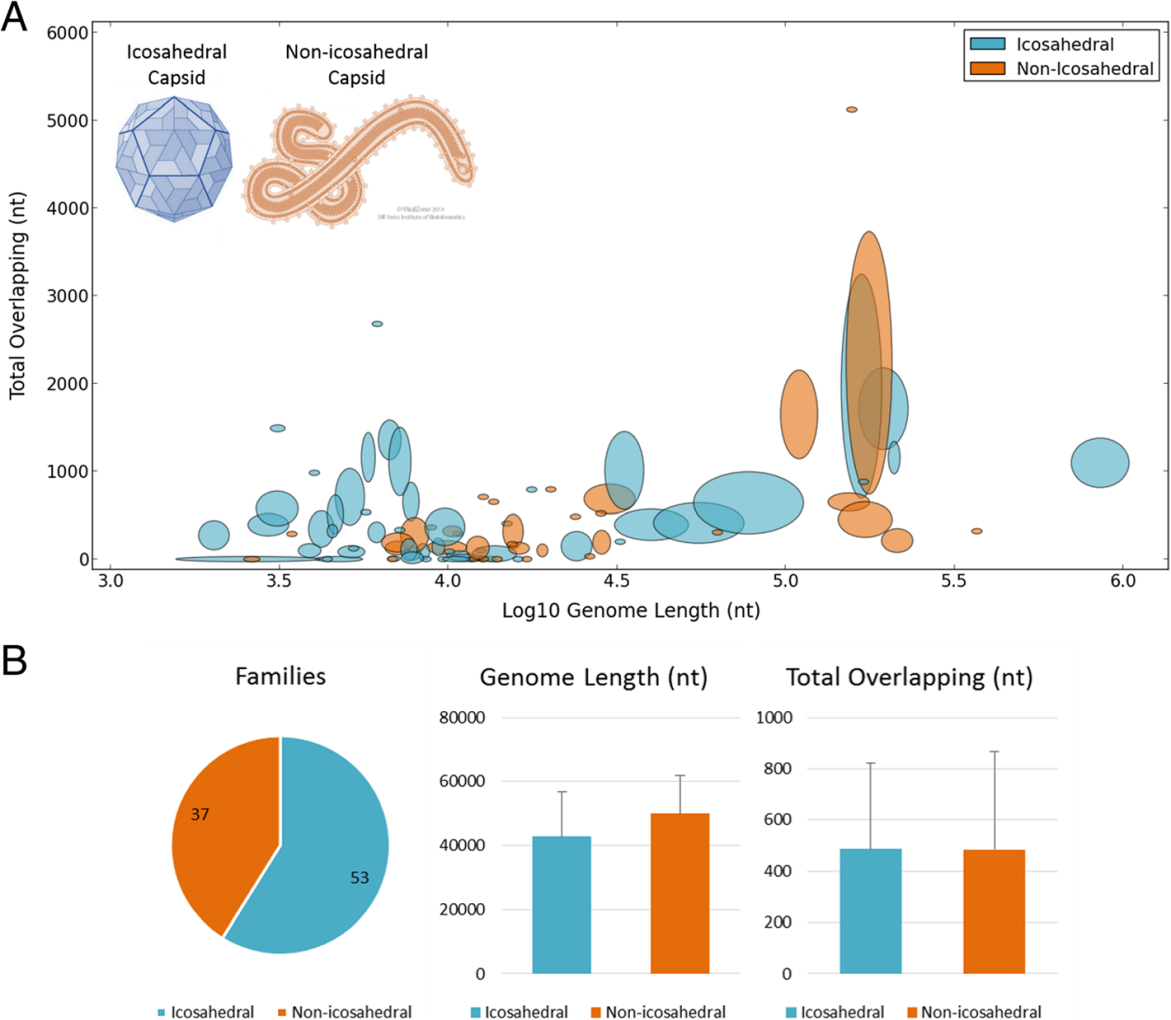
Overlapping amount and genome length are not associated with virion shape. **a** Showing the same analysis as in Fig. 2a with a different color scheme that highlights the partition between icosahedral and non-icosahedral viruses. Both classes are distributed all over the space. **b** A quantitative summary of the 90 families in the scatter plot (37 icosahedral and 53 non-icosahedral), showing the overall statistics of the two viral classes in family resolution. The two classes show similar values, in terms of both average and standard deviation

It had been claimed that icosahedral viruses have more overlapping, as a mechanism for overcoming the unique physical constraints imposed by their capsid shape [34, 36]. To test this claim, we considered the association between the physical shapes of icosahedral or non-icosahedral viruses to the phenomenon of overlapping. We revisited the viral landscape (as shown in Fig. 2a) and highlighted the partition between these two structural viral classes (Fig. 4a). Figure 4b provides a quantitative summary of these results. It is clear that the two classes are almost indistinguishable in terms of overlapping and genome length, both showing very similar values and patterns. We conclude that, globally speaking, virion shape does not present a meaningful relation to overlapping.

### Genome length is not constrained by capsid volume

In order to further understand whether there exist physical constraints that limit the evolution of viruses, thus driving for their exceptional rates of overlapping (Fig. 1b), we analyzed different aspects of capsid volumes.

**Fig. 5:**
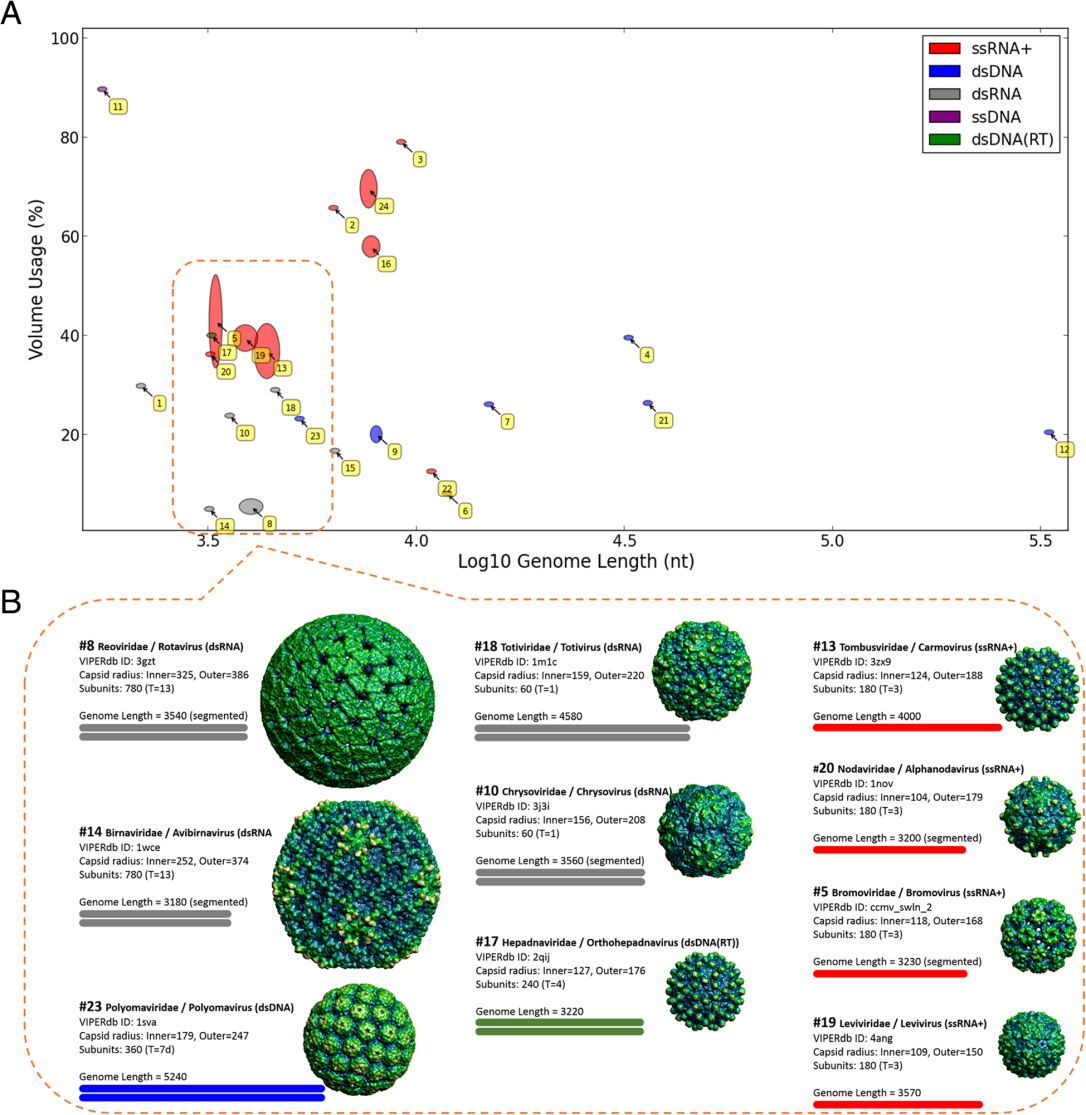
Capsid volume usage is often low and varies significantly among viral families. **a** A scatter plot demonstrating the volume usage (in %) with respect to genome lengths. Only the X-axis is in log scale. The ellipses were created by first calculating the volume usage percentage for each genus separately, and then drawing the families by the distributions of these values. The analysis covers all icosahedral viruses that are associated with detailed 3D information. There are 24 such icosahedral families: 1 – Partitiviridae, 2 – Tymoviridae, 3 – Dicistroviridae, 4 – Rudiviridae, 5 – Bromoviridae, 6 – Togaviridae, 7 – Tectiviridae, 8 – Reoviridae, 9 – Papillomavirida, 10 – Chrysoviridae, 11 – Circoviridae, 12 – Phycodnavirida, 13 – Tombusviridae, 14 – Birnaviridae, 15 – Cystoviridae, 16 – Caliciviridae, 17 – Hepadnaviridae, 18 – Totiviridae, 19 – Leviviridae, 20 – Nodaviridae, 21 – Adenoviridae, 22 – Flaviviridae, 23 – Polyomaviridae, 24 – Picornaviridae. Spearman's rank correlation is not significant: ρ = −0.17, *p*-value = 0.42. **b** An arbitrary sample of 10 families presented in (**a**), demonstrating the proportions of their capsid and genome sizes, from which the volume usage is derived. A single genus was chosen to represent each family, illustrating its capsid (with surface images from VIPERdb) and genome size (showing a bar proportional to its length that also displays the number of strands, and using the color of the relevant viral group). The radii of the capsid images are proportional to their outer radius (although it's the inner radius that determines the volume usage; both are written). Additional structural details (number of capsid subunits and T number) are also shown. The representative genus of each family was chosen by uniform rule - the one with the largest inner radius. This rule also applied for the displayed VIPERdb record

We used VIPERdb [44], the most exhaustive resource for accurate structural data of viruses that provides detailed structural measures for icosahedral viruses. We calculated the volume usage of viruses (see Methods). We found that there is no correlation (ρ = −0.17, *p*-value = 0.42) between the genome length and capsid volume usage among all tested icosahedral families (Fig. 5a). The volume usage varies significantly between different viruses with no apparent pattern, and many viral families (also the very small ones) seem to be far from an optimal use of their apparent capsule space. These results remain valid also when replacing the 24 representing families with the 37 genera that compose them (Additional file 2).

Table 1 provides a natural partitioning of the data presented in Fig. 5. Although double-stranded viruses have, in average, only half the volume usage of single-stranded viruses (24 % instead of 49 %), both lack a correlation between volume usage and genome length. We further tested the sensitivity of the calculation towards families with segmented viruses. When repeating the analysis with the exclusion of all segmented viruses (ending up with 18 families instead of 24), we observed only a minor effect on our global analysis (not shown).

**Table 1:** Volume usage in single- vs. double-stranded icosahedral families

	Number of Families	Average (%)	Standard deviation (%)	Spearman’s rank correlation between volume usage and genome length	
				ρ	*p*-value
Single-stranded viruses	11	49	25	−0.3	0.37
Double-stranded viruses	13	24	10	0	1
All viruses	24	35	22	−0.17	0.42

Eventually, we tested the assumption that icosahedral viruses are unlikely to change their size throughout evolution [22, 46]. The classification of viral genera into families, which are evolutionary related, provided us with the opportunity to measure the variation of capsid volume within families as a derivative of the extent at which viruses may adjust their capsid size with respect to their genome length.

Table 2 summarizes the variation of capsid volume inside families, with respect to capsid dimensions. It relies on atomic structural data in VIPERdb. In order to quantify variation we used the coefficient of variation (CV) statistical measure calculated individually for each family with respect to its genera. Table 2 summarizes 40 genera in 13 families. Only families for which sufficient structural data was available are included (at least two genera per family). The results of this analysis demonstrate that a physical variation exists inside icosahedral families (16 % and 20 % on average for inner and outer volumes, respectively). In many instances the differences between the inner and outer volumes are substantial. For these instances, the default estimate of of virus size [47] that is often used is misleading.

**Table 2:** Volume variation within icosahedral families

Family	Genera	Inner capsid volume CV^a^	Outer capsid volume CV^a^
Single-stranded viruses			
Bromoviridae	2	0.4	0.16
Caliciviridae	4	0.03	0.1
Comoviridae	2	0.11	0.02
Leviviridae	2	0.03	0.04
Parvoviridae	3	0.11	0.48
Picornaviridae	5	0.1	0.84
Tetraviridae	2	0.04	0.06
Tombusviridae	3	0.19	0.2
Avgerage	2.88	0.13	0.24
Double-stranded viruses			
Papillomaviridae	2	0.11	0.06
Partitiviridae	2	0.06	0.01
Podoviridae	4	0.04	0.03
Reoviridae	7	0.4	0.31
Siphoviridae	2	0.49	0.33
Avgerage	3.4	0.22	0.15
Overall average	3.08	0.16	0.2

^a^ *CV* coefficient of variation, defined as the ratio of the standard deviation σ to the mean μ

## Discussion

During our work we attempted to uncover broad unified principles that apply to most viruses. Finding global trends that apply to all viruses requires a careful unbiased approach. Obviously, our work is limited to the current coverage and classification of the viral world (Additional file 3). Due to the importance of some viruses for human health (e.g., HIV, HBV), fishery and agriculture, some viruses have been studied much more extensively than others. The outcome is an expansion in the number of reported species and genera in those well-studied families. By discussing the viruses at the family resolution, we overcome such imbalanced representation.

A major consideration in our study was to include all known viruses, using an unbiased representation. As a result, we were able to detect trends spanning three orders of magnitude in genome length, with only few outliers. Such interesting outliers (Fig. 2a) include the “giant viruses” Phycodnaviridae and Iridoviridae, described in the literature as very unusual in many aspects, to the extent that it was suggested to reclassify them as a new branch in the domains of life [48, 49].

From evolutionary perspective, gene overlapping comes with a great price. Two functional proteins that overlap significantly (and non-trivially; Fig. 1a) lead to evolutionary conflicting trends, a phenomenon that was addressed as ‘constrained evolution’ [50]. In order for a random missense mutation in an overlapping region to remain in the population, it must be beneficial for both ORFs (or beneficial for one of them and neutral for the other). Since such an event is very unlikely, overlapping induces a great burden over the evolvement of any organism [51].

We confirmed the existence of a significant negative correlation between genome length and overlapping rate (ρ = −0.59, *p*-value = 6.97·10E-9, Fig. 1b). Previous studies have interpreted this strong negative correlation as evidence in favor of the compression theory [34] over alternative explanations (see Background). However, by including families without any overlapping (13 families) the correlation becomes significantly weaker (ρ = −0.29, *p*-value = 0.0047). More critically, the observed correlation is merely a by-product of the way overlapping is calculated (see Results). It is governed by the data representation as a relative value rather than by absolute nucleotide counting. Instead, we found an overlooked pattern – the absolute amount of overlapping is highly bounded throughout all viruses, ranging in their length from ~1500 to over 1 million nucleotides (Fig. 2). The compression theory does not provide an explanation to this finding. The compression theory seems especially unlikely in view of our observations in large viruses. For example, the Baculoviridae family has four genera, with an average of 111,260 nt genome containing 122 genes and 1647 overlapping nucleotides. Theoretically, two extreme scenarios could have been accounted for such overlapping: (i) minor overlapping events spread over many genes; (ii) substantial overlapping events over a small subset of genes. If compression were the main driving force for overlapping, the first strategy would be evolutionary preferred, as small overlapping events are not expected to impose significant evolutionary constraints. However, it turns out that the Baculoviridae family leans more towards the second strategy. Specifically, this family has (on average) 2.5 significantly overlapping (300+ nt) genes. Moreover, the entire overlapping in this family accounts for less than 2 % of its genome length, so it is unlikely that overlapping contributes significantly to compression. This argument can be generalized to most families of large viruses (Figs. 2a and 3). Eventually, the relative perspective and the use of an inclusive definition of overlapping led to the notion that viruses have exceptional amounts of overlapping compared to other organisms (that have orders-of-magnitude larger genomes). A systematic approach had been applied to remove many of the spurious ORFs [52].

Instead of the compression theory, we suggest that the observed pattern of overlapping revealed in this study is in accord with the theory of gene novelty (e.g., [2]). According to this theory, random mutations sometimes introduce a legitimate start site on top of an existing coding gene, resulting in a new reading frame overlapping it. In fact, overlapping seems to be practically the only plausible way for viruses to increase their gene repertoire due to their compact genome organization (i.e., lack of introns or substantial intergenic regions). All other cases of gene gains must involve major genomic rearrangements or host genome contribution (e.g., gene duplication, recombination).

As the gene novelty theory predicts, it has been confirmed that many overlapping events involve a young (novel) gene coupled with an old well-founded partner [2]. Moreover, the signature of purifying selection has mostly been found in the older of the two. For example, in the Hepatitis B virus, purifying selection is evident in only one of the paired genes [50]. Proteins that originate from OGs are characterized by short sequence, enrichment in disordered regions, and unusual amino acid composition [37]. These results apply to all conformations of non-trivial overlapping. A strong argument in favor of the gene novelty theory comes from the species-specific nature of OGs (e.g., [53]). Novel OGs are generally orphans, lacking any remote homologs, unlike their older partners [25].

Unlike the compression theory that could not explain the bounded amount of overlapping and other patterns observed in Figs. 2a and 3, the theory of gene novelty provides a straightforward explanation by illustrating overlapping as a transient condition. Specifically, a significant overlapping event is not expected to last for long, due to the constant evolutionary burden imposed by it. Either one of the OGs will evolve on the expanse of the other, until it fades away, or, alternatively, they will become uncoupled (e.g., by gene duplication). Furthermore, by seeing gene novelty as the major driving force for overlapping events, it is anticipated that at any given point in time, only a small number of novel genes will be introduced to cope with the changing environment. Assuming that viruses are specified by non-redundant indispensable gene composition, the number of gene exploration events they could tolerate simultaneously is limited. This evolutionary pressure will lead to a bounded number of OG in all viruses, and it should depend very little on their genome length, as illustrated throughout our study. This observation supports the need for a limited exploration for viruses at any length, at any evolutionary window. The age and stability of novel ORFs is likely to be dependent on the specific viral family dynamics (e.g., [54].

Our reservation from the compression theory as the main evolutionary force driving for gene overlapping in viruses does not contradict the strong tendency of viruses to be small. Viruses are indeed highly compact, in the sense of having a minimal amount of unused regulatory regions and intergenic regions [55] with some exceptions [56], and that viral proteins are often shorter versions that converged toward simpler domain compositions [12]. We simply claim that overlapping is not a significant factor in the compression of viral genomes. From the perspective of information theory, overlapping does not increase the amount of information in a genome (as measured in bits of entropy), but only partitions it among a larger set of genes, allowing more genes with less information in each. This dictates novel OGs to be poor in information, lacking complex structure and function and capable of tolerating high number of mutations. It was shown that most novel OGs are nonstructural and carry simple function [25, 33, 37].

Although information-poor, novel OGs with simple unstructured protein products may still be beneficial for the virus by filling various simple functions, mostly by affecting the host cell. Such functions may include preoccupying the cellular systems of the host [12], overloading the immune system [57], activating ER stress [58], causing autoimmune diseases by a molecular mimicry [59], leading to ubiquitination, and more [60, 61]. It is reasonable to assume that a virus needs only a limited number of such novelties at any given point in time, which is another potential explanation for the limited number of OGs in viruses.

It was also claimed [34] that icosahedral viruses have more overlapping than non-icosahedral, because the capsid size of the former is less flexible and unable to grow continuously, consequently these viruses are not capable of increasing their genome length. Our results dispute these claims. First, the pattern of overlapping and genome length is similar in both icosahedral and non-icosahedral viruses (Fig. 4). Moreover, if there is any difference in the variance of genome length inside families between these two classes, icosahedral viruses are in fact the ones with a slightly higher variance, suggesting that they are indeed capable of increasing or decreasing their genome length. It may still be that the higher variance observed in icosahedral families is merely a bias caused by the fact that an icosahedral family has more recorded genera on average (4.6 instead of 2.7).

Are icosahedral capsids unable to continuously change along evolution? Although changing the T number would result a major change in the capsid size, it might indeed be possible to slightly change the size of each subunit composing the capsid. Indeed, a variance in both the inner and outer capsid volumes exists among the genera of icosahedral families (Table 2). Our structural results undermine the common claim that the alleged compression requirement of viruses is driven by physical size constraints imposed by a limited space in their capsid. Figure 5 shows a great variance in volume usage among families (distributed with no apparent pattern), suggesting that physical space is probably not a significant constraint for viral evolution, as many viruses, even small ones, use only a small fraction of the volume available for them. The observation that the volume usage of single-stranded viruses is significantly higher than that of double-stranded (49 % vs. 24 % on average, Table 1) remains unexplained. However, in some families the viruses are packed with additional proteins that are essential for the infectivity (e.g., Vif protein in HIV [62]). Others carry replication (polymerase) or packing proteins. The volume usage estimation ignores the contribution of any proteins that might be packed inside the virion, whether produced by the virus or the host. In most instances these proteins occupies a minor fraction of the inner volume. Eventually, there are different mechanisms for packing viral genomes inside a capsid [63]. In bacteriophages, the packing of the dsDNA is essential for a successful ejection during infection. On the other hand, effectible packing and compressing single-stranded genomes (RNA and DNA) is based on electrostatic interaction of the capsid proteins with the nucleic acids negative charges [64].

One would quickly find out that it is a lot easier to make hypotheses about the entire viral world rather than proving them. This complex behavior of volume usage raises concerns about the interpretation of a recently reported study showing a strong linear correlation between the logarithm of viral genome lengths to the logarithm of their capsid volumes [47]. It was originally interpreted that a strong polynomial relationship exists between these two variables (since log *y* ≈ *A* log *x* + *B* suggests *y* ≈ *e*^*B*^*x*^*A*^) and that “virion sizes in nature can be broadly predicted from genome sequence data alone”. Although we obtained a similar linear correlation (R^2^ = 0.77, *p*-value = 1.49·10E-8; Additional file 4), our analysis does not support a polynomial model. We have demonstrated a great variation in volume usage, with most viruses in the range of 20–80 % (Fig. 5), meaning that predicting the capsid size from genome length cannot be accurate. Indeed, the suggested polynomial model contains errors of up to an order of magnitude [47]. Furthermore, this polynomial model is not robust to natural partitioning of the data. For example, the results of the linear regression change dramatically from a coefficient of 0.9 in double-stranded to 1.58 in single-stranded viruses (where the coefficient for both is 1.13). Obviously, these give very different polynomial models, *y* = *C*_1_*x*^0.9^ vs. *y* = *C*_2_*x*^1.58^, suggesting that over-fitting is involved.

As our results rely on a statistical analysis, we do not expect them to apply to every single family, nor to all possible subsets of the data. It is likely that special viral taxa do not follow some of the general trends we found. We share our raw data and the computational code to assist researchers to further study this subject (see Additional files 5, 6, 7 and 8).

Understanding the driving forces and constraints that govern viral evolution becomes highly relevant in view of epidemic episodes and outbreaks in recent years (e.g., [65]). The task of developing sustainable antiviral treatment strategies and sophisticated viral-based delivery systems heavily depends on it [66, 67].

## Conclusions

We have shown that the negative correlation that exists between genome length and overlapping rate in viruses is merely a side effect of a broader phenomenon: the absolute amount of gene overlapping is strictly bounded across the entire viral spectrum (Fig. 2). We have also demonstrated that icosahedral and non-icosahedral viruses are indistinguishable in their patterns of gene overlapping, and that icosahedral viruses often utilize only part of the capsid volume available to them. Furthermore, icosahedral viruses seem capable of changing their capsid volume along evolution.

All these pieces of evidence suggest against the common theory that viral gene overlapping has a role in genome compression. Instead we suggest that gene novelty and evolution exploration better explain our findings. Gene overlapping can be a convenient mechanism to introduce new reading frames on top of an already compact genome, providing an easy expansion of a virus’s gene repertoire, thus allowing it to cope with the changing environment and endure the combative virus-host coevolution race.

## Methods

### Data and resources

We used two main data sources: ViralZone ([45]; http://viralzone.expasy.org/) and VIPERdb ([44]; http://viperdb.scripps.edu/). ViralZone has been used for a taxonomical categorization of the International Committee on Taxonomy of Viruses (ICTV). All viral species are classifies to replication groups, families, genera and species (see Additional file 3). It is linked to genomic data, through reference genomes from NCBI [68]. In addition, when structural data could not be found at VIPERdb for certain viral families, we also searched inside ViralZone pages for information about their icosahedral T numbers. Specifically, the T number information has been used to distinguish between icosahedral and non-icosahedral families. We assumed that a family is icosahedral if and only if it appears in VIPERdb or has a T number in ViralZone.

From VIPERdb we extracted capsid structural data, specifically the radiuses used for all the volume analyses. VIPERdb also classify the records by families and genera. We used this classification in order to match between ViralZone and VIPERdb records, providing us with both genomic and structural data for the common genera that appear in both resources.

VIPERdb document all the solved structures of icosahedral capsids (linked to its PDB record [69]). Each genus might have dozens of different records, several of the records represent mutated version of capsid proteins. In this study we were obviously interested in naturally occurring infective viruses. We thus combined all VIPERdb records sharing the same genus, ending up with a single record for each genus. When multiple values were available for a certain genus, we took the maximal value to represent the genus. Using this protocol we overcame the cases in which the capsid subunits collapse inwards (as often happens in mutated viruses) forming a shape incompatible with a proper natural capsid capable of containing the viral genome [70]. For example, the Mastadenovirus genus (of the family Adenoviridae in the dsDNA group) has ten records in VIPERdb, with inner radius values ranging from 41 to 326 Å. These radii belong to an artifact of an empty capsid and a natural infective virion, respectively. The inner radius of infective Mastadenovirus virions ranges from 311 to 326 Å. We merged these ten records into a single record representing the Mastadenovirus genus, whose inner radius was set to be 326 Å. A similar pattern occurs in most genera.

In order to retain maximal objectivity, it was crucial that the entire process of data extraction would be automatic, without any local decisions being made for specific records. For this reason all the data was extracted from the different databases (ViralZone, VIPERdb and NCBI) using unbiased downloading protocols and the analysis was performed on the entire set of records. Note that when we removed outliers it was merely for the sake of figure visibility. We clearly indicate the removed outliers in the figures’ caption and in the statistical analysis.

After extracting the data, we ended up with full taxonomic and genomic information of 352 genera in 93 families taken from ViralZone and its NCBI links. This number is slightly lower than the 420 genera reported in ViralZone (April 2015), as the missing genera did not have a complete reference species. We processed 419 VIPERdb records, which were grouped into 68 genera in 37 families. For 43 genera in 28 families we had records from both ViralZone and VIPERdb. This set of 28 families was applied for volume analyses (see “volume calculations” section).

All the data we extracted is available as additional files submitted with this paper in CSV format (Additional files 5, 6 and 7). These files contain more fields and properties that can be useful for a follow up research. We share our Python code, which contains a handy framework for analyzing this data (Additional file 8).

### Taxonomy and representative selection

Different families might dramatically vary in the number of recognized genera they cluster together (e.g., the Picornaviridae family has 23 reference genera in ViralZone, while many other families have only 1). In this study we sought to conduct an unbiased statistical analysis. Thus, we conducted most of it at the family resolution, giving an equal weight to all viral families, regardless of the number of genera and species they might have.

For each variable involved in the analysis, we took the family’s value to be the average among all of its genera. When calculating Spearman’s rank correlation, for example, the samples used for the statistical test were actually the average values of each family. Yet, in order to also show the variety that might exist within families, we drew each family as an ellipse. The ellipse’s center corresponds to the average value of the family’s genera, and its width and height correspond to the standard deviations of its genera with respect to each of the two studied variables.

Throughout this study we ignored the variation within genera, taking the value of each genus to be the maximum among its species, doing so for each property separately. For example, the genome length of a genus was determined by its species with the maximal genome length.

### Overlapping measurements

We define the amount of overlapping in a genome to be the number of nucleotides (nt) involved in a non-trivial gene overlapping events. A trivial overlapping event is when the two genes overlap but the same reading frame (and strand) is used (Fig. 1a). The majority of overlapping instances in viruses are trivial, where the end product is an extended version of the same protein with alternative start or stop sites (obviously, this leads to more than one protein with the same amino-acid sequence coded by the overlap region). Trivial overlapping lacks all the interesting evolutionary implications, hence we removed it of the analysis. Also, whenever referring to genes, only protein-coding regions are considered. It follows that overlapping amount, which is given in nucleotides, can immediately be translated to amino acids (i.e., 3 nt to 1aa).

Overlapping rate is defined as the relative part of the genome involved in overlapping (i.e., the amount of overlapping divided by the genome length). We define an overlapping event to be significant (coined SOG), if it involves at least 300 nt from both OGs. As we have demonstrated, our results are not sensitive to this exact threshold, but having a threshold is crucial (see Results). Recall that every overlapping event involves at least two genes, so when talking about the number of genes in a genome involved in overlapping, the number of overlapping events is usually only half that number.

### Volume calculations

Most volume analyses were limited to the 28 viral families for which we had both high quality genomic data from NCBI (linked from ViralZone) and capsid structural data (from VIPERdb). We defined the volume usage of a virus to be the ratio of its genome volume to the volume of its capsid. Some genera resulted an apparent volume usage that exceeds 100 %. These are capsid shapes that resulted from artificial mutated proteins, as an in-vitro assembly of a capsid without its genome often results in a collapsed shape. We filtered such genera out of the volume analyses, ending up with 24 families that had at least one proper genus.

Icosahedral solids are roughly spherical, so we calculated their volumes by the formula of a ball’s volume: $V=\frac{4}{3}\pi {r}^3$, where r is the capsid’s inner radius, as provided by VIPERdb. Genomic volumes were calculated assuming that double-stranded DNA (or RNA) molecules are roughly cylindrical with a ~20 Å diameter and a distance of ~3.4 Å between adjacent nucleotides in the backbone [71], yielding $V=\left(3.4\times L\right)\times \pi \times {\left(\frac{20}{2}\right)}^2\approx 1,068L$, where L is the genome length (in nt). For single-stranded genomes we took half that volume (i.e., *V* ≈ 534*L*). This calculation ignores higher-order conformations of the genomic material, making it only a lower bound to the true genomic volume. Despite the limitation in calculating the usage of the capsid volume, we would still expect to see a uniform volume usage for the different families if the idea that viruses utilize their available space were correct. Hence, despite this limitation, our results still suggest that many viruses do not fully utilize their available capsid volume.

Another complication in calculating genome volume arises from segmented viruses (see background). It was shown that different particles most likely have only a subset of the segments [19], so we calculated genome volume based on the length of the longest segment. As mentioned in the Results, our analysis was not sensitive to the exclusion of all segmented viruses.

## Reviewers’ comments

**Reviewer 1:** Arne Elofsson has requested no changes. He referred to the manuscript as an elegant work.

**Reviewer 2:** David Kreil comments: The manuscript on “Gene Overlapping and Size Constraints in the Viral World” by Brandes and Linial exploits well-curated resources that comprehensively classify the viral world, collecting annotated genomes, and adding high-resolution structures where available, in order to conduct an unbiased systematic survey of about 400 viral genera in about 100 families. The focus of their examination is an improved understanding of possible mechanisms driving the evolution of viral genomes; in particular the question whether capsid size can explain genome length and a pressure for overlapping genes. In fact, the clear analysis presented in this work unambiguously favours one of the several competing theories for the mechanism behind gene overlaps, namely that the overlaps result from novel genes being introduced ‘on top’ of established genes. The authors’ analysis is exemplary in rigour and comprehensiveness, and the laid out data and arguments are highly convincing, making this work a landmark contribution in the field. The co-publication of data and source code is highly commendable. The paper would still benefit from the below suggested revisions to figures, and I hope that the authors can use the opportunity to further strengthen the presentation of the manuscript in revision.

Response: *We thank the reviewer for nicely summarizing the highlight of our work. We took the liberty to number the minor comments of the reviewer.*

1. Figure 1.a. The examples shown are important, yet the illustrations are a little confusing. I think it would help if the legend could be extended, e.g., to explain the colour coding of the ORF/frames.

*Response: We included the missing information in the legends of Fig.**1**a as requested.*

2. Figure 1.b. To facilitate an interpretation of the variance shown as size of the ellipses, can you somehow also indicate the size of each family (perhaps via the line width?). Please clarify in the legend whether the area or the height of the ellipses is indicative of the variance (the legend currently reads “size”). As the variance is the standard deviation squared, using area of the ellipse to reflect the variance would seem the more appropriate choice [this analogously applied to the other ellipse diagrams as well].

*Response: The reviewer questioned the size of the ellipses. Indeed as the reviewer noted, the area of the ellipse captures best the variance, as the width and height indicate the standard deviation. We modified the legend of this figure to clarify this. The variation of a family is defined by the values of the genera within that family. The list of families and their genera is found in the relevant supplementary tables.*

3. Figure 3.b. Please also give the genome sizes for the two filtered families in the legend.

*Response: As requested, this information was added in the legend of Fig.**2**a. Since the same families are always the outliers, we saw it unnecessary to repeat this information in all of the figures.*

4. Figure 4.b. For completeness, please also quantify “similar” means and standard deviations by including the non-significant p-values for tests of differences.

*Response: Actually, we tried to avoid the (overestimated) p-values. The p-values are sensitive to small differences that often look significant, if enough data points are involved.*

5. Figure 5.b. For clarification: “The sizes of the capsids…” -->“The radius of the capsid images…”; I would also suggest: “rigid rules” -->“a uniform rule”. I do not understand the second half of this sentence: “as well as the displayed… record” - can you please rephrase to clarify?

*Response: The sentences were rephrased as suggested.*

6. Regarding Additional file 2: Do the other observations of this paper also hold at the genus level?

*Response: Throughout this study we focused on family rather than genus level of resolution, which we argue to be the appropriate resolution to look at. The information in Additional file**2**was added to further substantiate the finding in Fig.**5**.* Figure *5**included only 24 families. The addition of data-points at the genus level (total of 37) allowed to overcome the somewhat limited number of data-points of the analysis.*

## Additional files

Additional file 1: Supplemental figure – The observation that the number of significantly overlapping genes is bounded is only mildly affected by the choice of thresholds. Two plots presenting the same analysis as in Fig. 3a, where only significantly overlapping genes (SOGs) are considered, but with different thresholds for the definition of what is considered significant: 100 nt (A) and 50 nt (B). Only the X-axis is in log scale. Filtered out 3 outlying families (Nimaviridae, Phycodnaviridae and Iridoviridae with 461/472 of 532, 200/225 of 505 and 70/75 of 186 SOGs, respectively), leaving 90 shown families. Spearman rank correlation: ρ = 0.16, *p*-value = 0.13 (A) and ρ = 0.33, *p*-value = 0.0015 (B). (PNG 264 kb) Additional file 2: Supplemental figure – The lack of pattern in volume usage is evident also at genus resolution. A plot showing the same analysis as in Fig. 5, but in genus resolution. Instead of the 24 families used in Fig. 5, we now consider the 37 genera composing them, giving each an equal weight in the analysis. Spearman’s rank correlation is insignificant: ρ = 0.1, *p*-value = 0.56. (PNG 87 kb) Additional file 3: Supplemental figure – Viral taxonomy. (**A**) The evolvement of the viral world classification in over 40 years, according to the International Committee on Taxonomy of Viruses (ICTV, 2013). As for 2013, there are 2827 recognized species in 455 genera in 103 families. Most of the time, the number of recognized species has been growing steadily, but in 1998 it dropped from 2370 to 1551, due to reconsideration of former classifications. (**B**) A classification example, showing the Zaire ebolavirus species, which is a member of the Ebolavirus genus in the Filoviridae family of the ssRNA- group. (PNG 189 kb) Additional file 4: Supplemental figure – There exists a strong linear correlation between the logarithm of genome volumes to the logarithm of capsid volumes in icosahedral families. A scatter plot showing the relationship between genome volumes to inner capsid volumes in viral families. Both axes are in log scale. 24 families are shown (the same families as in Fig. 5). Linear regression: R^2^ = 0.77, *p*-value = 1.49·10E-8, y = 1.13x - 0.3. Spearman’s rank correlation: ρ = 0.83, *p*-value = 4.15·10E-7. Although the correlations are very significant, it should be reminded that the data is presented in a double log scale. This presentation has a tendency to “flattening the data”, making regressions analyses better, and underestimating the errors. For example, the linear model predicts for the Reoviridae family (dsRNA) an inner capsid volume of 16 million Å^3, where in fact it has 85 million (more than a 5-fold difference). (PNG 88 kb) Additional file 5: ViralZone data. Contains all the taxonomical and genomic data we have used in this study, as taken from ViralZone and its references to NCBI. Rename this file to “viralzone.csv” in order to load it through our Python framework. (CSV 7614 kb) Additional file 6: VIPERdb clean data. Contains structural data about the capsids of icosahedral viral genera, as taken from VIPERdb after merging together records of the same genus (see Methods). Rename this file to “viperdb_clean.csv” in order to load it through our Python framework. (CSV 6 kb) Additional file 7: VIPERdb raw data. Contains structural data about the capsids of icosahedral viral genera, as taken from VIPERdb without any further processing. Rename this file to “viperdb_raw.csv” in order to load it through our Python framework. (CSV 63 kb) Additional file 8: Python code we have used during our analysis. This code allows not only to reproduce our research, but is also a handy framework for our data (Additional files 5, 6 and 7 of CSV format), which contains even more fields than we have used here. We strongly encourage researcher that are interested in further studying the topic to make use of our code and data. (RAR 13 kb)

## Abbreviations

OGs: overlapping genes
SOGs: significantly overlapping genes
CV: coefficient of variation
ss: single stranded
ds: double stranded
ORF: open reading frame
nt: nucleotides
