# Supplementary figures and images for: Gene overlapping and size constraints in the viral world
Source: Biol Direct. 2016 May 21;11:26. doi: 10.1186/s13062-016-0128-3 (PMC4875738; doi:10.1186/s13062-016-0128-3)

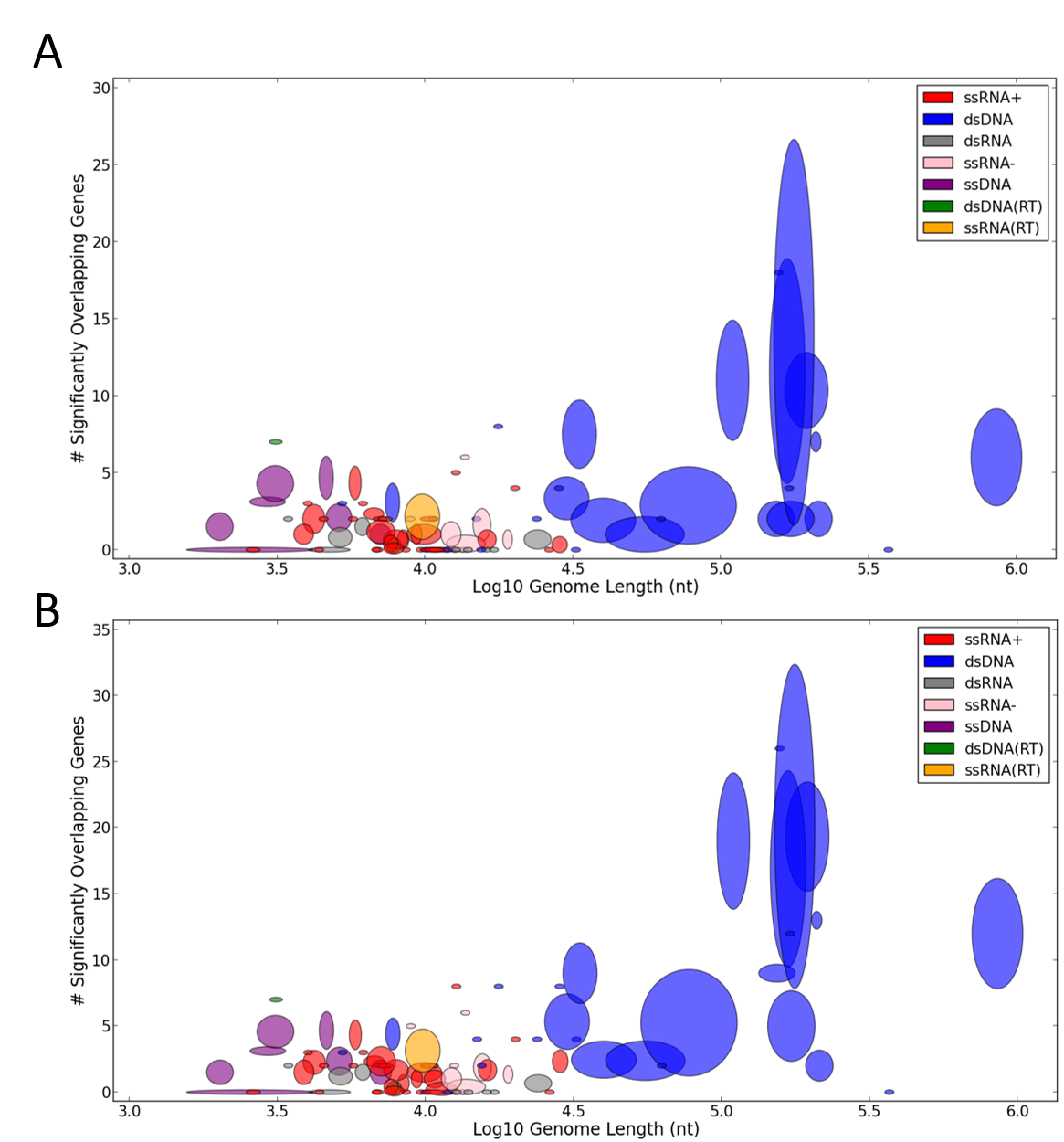

Supplement: Additional file 1: — Supplemental figure – The observation that the number of significantly overlapping genes is bounded is only mildly affected by the choice of thresholds. Two plots presenting the same analysis as in Fig. 3a, where only significantly overlapping genes (SOGs) are considered, but with different thresholds for the definition of what is considered significant: 100 nt (A) and 50 nt (B). Only the X-axis is in log scale. Filtered out 3 outlying families (Nimaviridae, Phycodnaviridae and Iridoviridae with 461/472 of 532, 200/225 of 505 and 70/75 of 186 SOGs, respectively), leaving 90 shown families. Spearman rank correlation: ρ = 0.16, p-value = 0.13 (A) and ρ = 0.33, p-value = 0.0015 (B). (PNG 264 kb) [file 13062_2016_128_MOESM1_ESM.png]

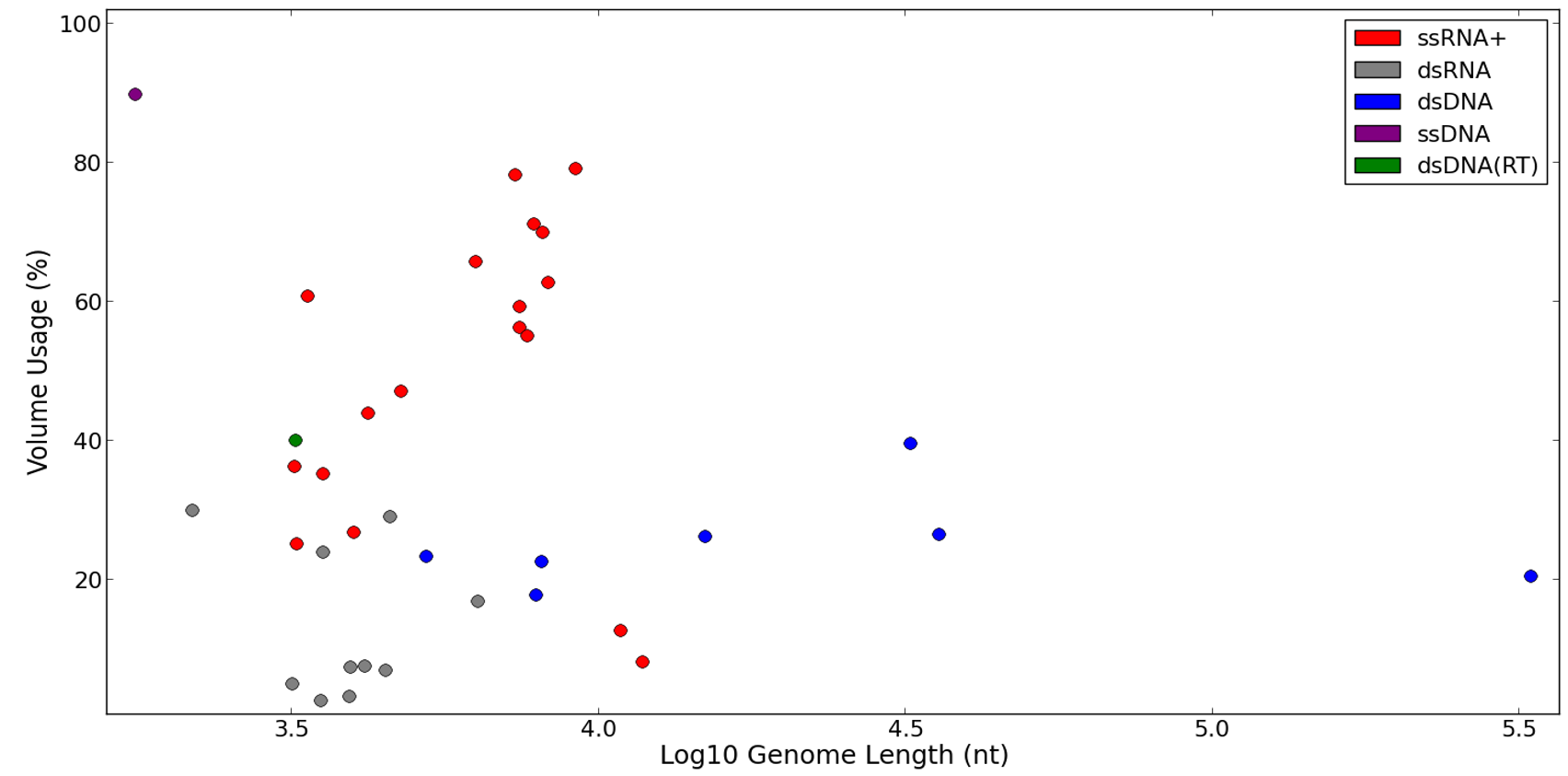

Supplement: Additional file 2: — Supplemental figure – The lack of pattern in volume usage is evident also at genus resolution. A plot showing the same analysis as in Fig. 5, but in genus resolution. Instead of the 24 families used in Fig. 5, we now consider the 37 genera composing them, giving each an equal weight in the analysis. Spearman’s rank correlation is insignificant: ρ = 0.1, p-value = 0.56. (PNG 87 kb) [file 13062_2016_128_MOESM2_ESM.png]

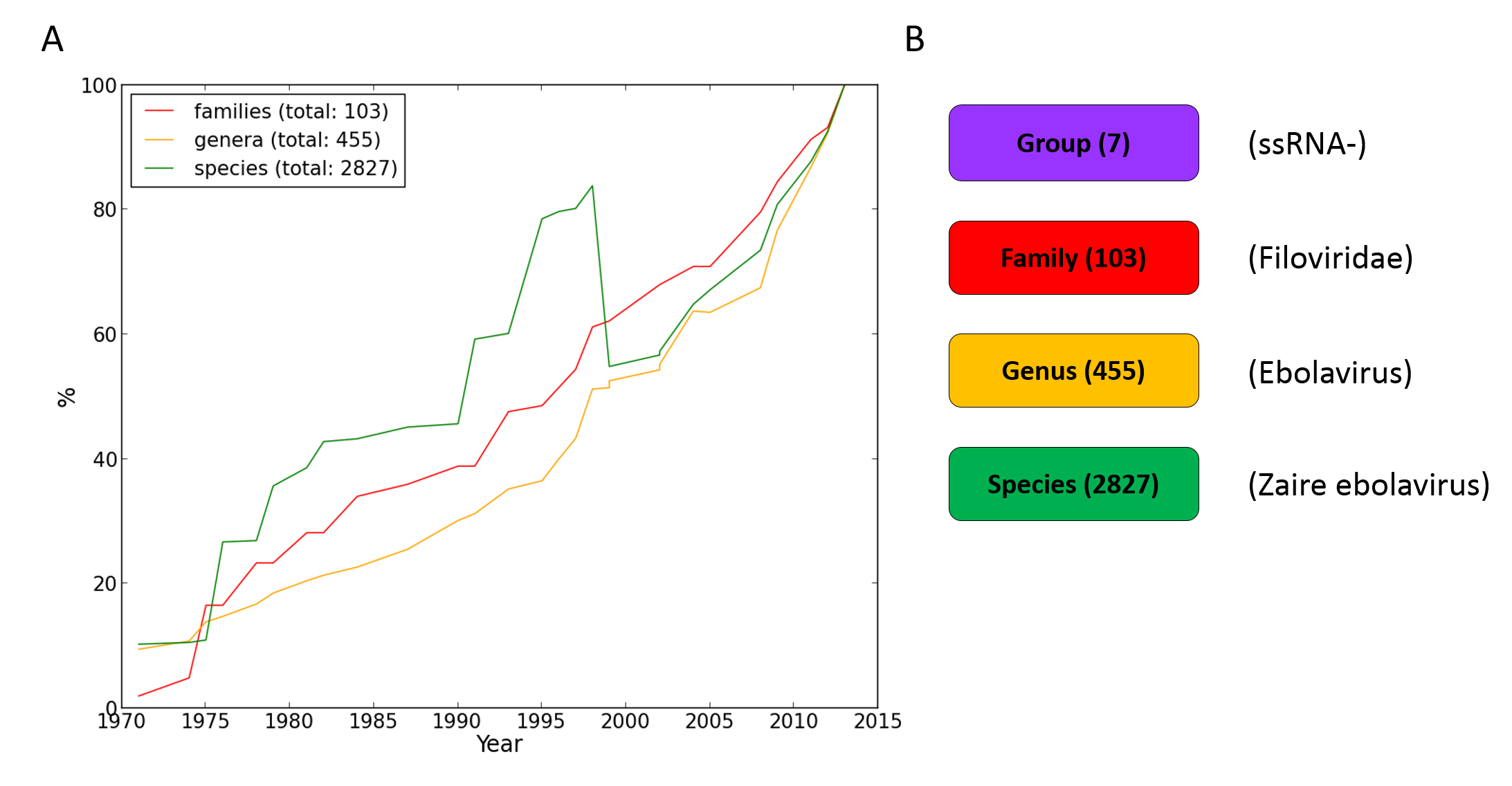

Supplement: Additional file 3: — Supplemental figure – Viral taxonomy. (A) The evolvement of the viral world classification in over 40 years, according to the International Committee on Taxonomy of Viruses (ICTV, 2013). As for 2013, there are 2827 recognized species in 455 genera in 103 families. Most of the time, the number of recognized species has been growing steadily, but in 1998 it dropped from 2370 to 1551, due to reconsideration of former classifications. (B) A classification example, showing the Zaire ebolavirus species, which is a member of the Ebolavirus genus in the Filoviridae family of the ssRNA- group. (PNG 189 kb) [file 13062_2016_128_MOESM3_ESM.png]

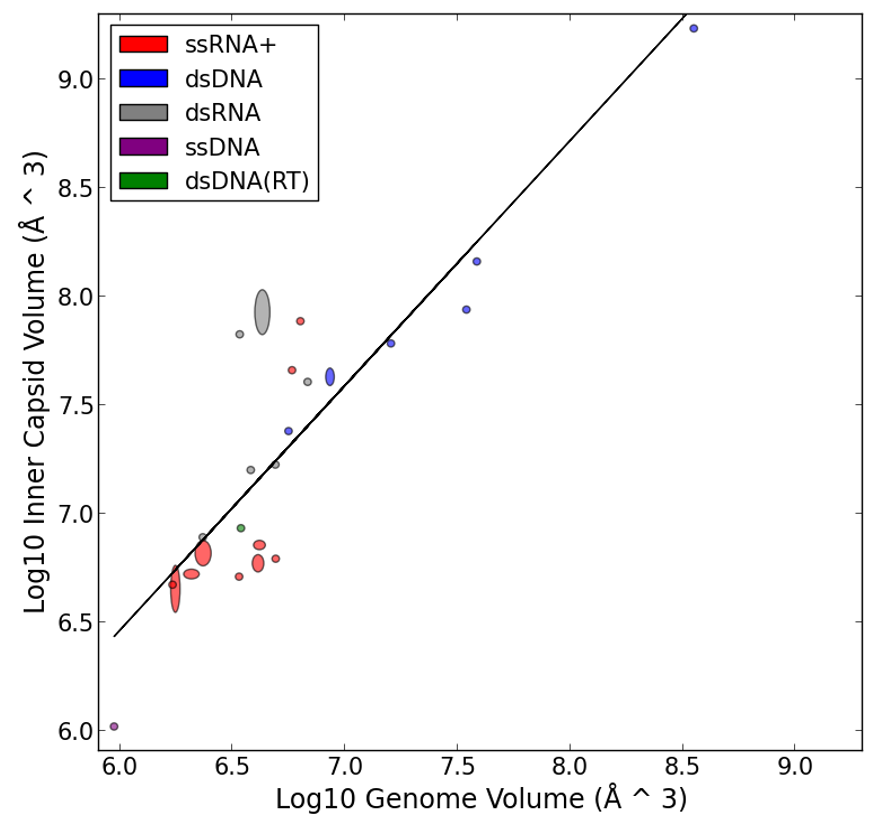

Supplement: Additional file 4: — Supplemental figure – There exists a strong linear correlation between the logarithm of genome volumes to the logarithm of capsid volumes in icosahedral families. A scatter plot showing the relationship between genome volumes to inner capsid volumes in viral families. Both axes are in log scale. 24 families are shown (the same families as in Fig. 5). Linear regression: R2 = 0.77, p-value = 1.49·10E-8, y = 1.13x - 0.3. Spearman’s rank correlation: ρ = 0.83, p-value = 4.15·10E-7. Although the correlations are very significant, it should be reminded that the data is presented in a double log scale. This presentation has a tendency to “flattening the data”, making regressions analyses better, and underestimating the errors. For example, the linear model predicts for the Reoviridae family (dsRNA) an inner capsid volume of 16 million Å^3, where in fact it has 85 million (more than a 5-fold difference). (PNG 88 kb) [file 13062_2016_128_MOESM4_ESM.png]
